# Supplementary material for: MetaRibo-Seq measures translation in microbiomes
Source: Nat Commun. 2020 Jun 29;11:3268. doi: 10.1038/s41467-020-17081-z (PMC7324362; doi:10.1038/s41467-020-17081-z)
Supplement: Supplementary file 10 — Supplementary Data 7 [file 41467_2020_17081_MOESM10_ESM.zip › File2/Confidence_High_Taxonomy/86412_out.krona.html]

Javascript must be enabled to view this page.

members
magnitude
magnitudeUnassigned
count
unassigned
taxon
rank

86412\_out

2

superkingdom
2
2

2
phylum
1239

186801
class
2

186802
2
order
1

SRS014979\_contig\_number\_4619

family
186806
1

genus
1730
1

142586
species
1

SRS019496\_contig\_number\_9297
